# What is the state of children’s participation in qualitative research on health interventions?: a scoping study

**DOI:** 10.1186/s12887-022-03391-2

**Published:** 2022-06-04

**Authors:** Jean M. Hunleth, Julie S. Spray, Corey Meehan, Colleen Walsh Lang, Janet Njelesani

**Affiliations:** 1 Division of Public Health Sciences, Washington University in St. Louis School of Medicine, 660 S. Euclid Avenue, St. Louis, MO 63110 USA; 2 National University of Ireland, Galway, Ireland; 3 University of Colorado School of Medicine, Denver, CO USA; 4 Department of Anthropology, Washington University in St. Louis, St. Louis, MO USA; 5 Loma Linda University Health, Loma Linda, CA USA; 6 New York University, New York City, NY USA

**Keywords:** Children, Health interventions, Childhood studies, Meaningful participation, Implementation research, Scoping review

## Abstract

**Background:**

Children are the focus of numerous health interventions throughout the world, yet the extent of children’s meaningful participation in research that informs the adaptation, implementation, and evaluation of health interventions is not known. We examine the type, extent, and meaningfulness of children’s participation in research in qualitative health intervention research.

**Method:**

A scoping study was conducted of qualitative published research with children (ages 6–11 years) carried out as part of health intervention research. Following Arksey and O’Malley’s scoping study methodology and aligned with the PRISMA-ScR guidelines on the reporting of scoping reviews, the authors searched, charted, collated, and summarized the data, and used descriptive and content analysis techniques. Ovid MEDLINE was searched from 1 January 2007 to 2 July 2018 using the keywords children, health intervention, participation, and qualitative research. Study selection and data extraction were carried out by two reviewers independently.

**Results:**

Of 14,799 articles screened, 114 met inclusion criteria and were included. The study identified trends in when children were engaged in research (e.g., post-implementation rather than pre-implementation), in topical (e.g., focus on lifestyle interventions to prevent adult disease) and geographical (e.g., high-income countries) focuses, and in qualitative methods used (e.g., focus group). While 78 studies demonstrated meaningful engagement of children according to our criteria, there were substantial reporting gaps and there was an emphasis on older age (rather than experience) as a marker of capability and expertise.

**Conclusions:**

Despite evidence of children’s meaningful participation, topical, geographical, and methodological gaps were identified, as was the need to strengthen researchers’ skills in interpreting and representing children’s perspectives and experiences. Based on these findings, the authors present a summary reflective guide to support researchers toward more meaningful child participation in intervention research.

**Supplementary Information:**

The online version contains supplementary material available at 10.1186/s12887-022-03391-2.

## Background

Qualitative approaches are critical for creating and adapting health interventions [1–3], and are increasingly used to inform pediatric health interventions, with parents, teachers, physicians, and other adult stakeholders participating in qualitative studies. While such adult participation can offer important perspectives on pediatric concerns, strong evidence in the field of childhood studies shows that research based solely on adult reports and perceptions risks misrepresenting children and their needs [4–7]. Childhood studies is an interdisciplinary field comprised of scholars from the social and humanistic sciences (e.g., anthropology, education, geography, psychology, sociology). The field is broadly interested in examining children and childhoods across cultural contexts and also in services and policies for children. Researchers working within childhood studies have well established that adults, including caregivers, do not always know what their children know, understand, or experience, and they might hold different understandings of children’s needs and subjective wellbeing [5, 6, 8–11]. This means that health intervention and implementation research also requires children’s perspectives. Further justifying their inclusion in intervention research, globally, children are increasingly considered key stakeholders, with the right to contribute to discussions on how services, including health services, are delivered to them [12]. However, the extent and nature of children’s participation in qualitative research that informs the adaptation, implementation, and evaluation of pediatric health interventions is not known.

Assessing the *meaningfulne*ss of children’s participation in qualitative health intervention research is particularly important in child research because children have historically been treated as objects, rather than subjects of or participants in health research, in part due to cultural constructions of children as incompetent or passive recipients of care [5]. This cultural inheritance has meant a lack of *participation ecosystem*—or the infrastructure, knowledge, institutional structures and cultural practices that facilitate participation—for supporting researchers in efforts to meaningfully include children in research [13]. Consequently, non-meaningful participation can result when methods are not sufficiently tailored to children’s needs and social position (e.g., power dynamics) or when children’s views are surveyed or interpreted superficially. While meaningful participation can take different forms, from youth-led participatory action research (where young people participate in planning, leading, implementing, and analyzing research) to young people contributing advice, ideas, or perspectives, to researcher-led projects, the aim of children’s meaningful participation in research is always to listen to children’s perspectives and experiences without tokenizing them or diminishing what they have to offer [14].

A focus on children in middle childhood (ages 6–11 years) is important because childhood researchers have shown that children in this age category contribute important insights on health and social topics, but that these perspectives are not always taken seriously by decision makers [5–7, 15, 16]. Also this age category is increasingly recognized as neglected in child health research, which focuses more on early childhood or on adolescence [17]. A prior scoping review evaluating the level of participation of young people under 25 in health interventions found that few studies supported higher levels of participation even given the proportionately higher representation of older age groups and alludes to a need to more deeply examine how participation diminishes with younger age [18]. We further justify our review as critical given the expanding emphasis on using qualitative research to inform programmatic and policy intervention work in pediatrics, and also because including children in qualitative research in *meaningful* ways, as the field of childhood studies has shown, often demands creative, flexible, and participatory approaches (for example, see [5–7, 15, 19, 20]). It also demands a specific researcher stance, whereby researchers view children as social actors within their environments and acknowledge generational power dynamics [8, 16, 21–26].

Given the above needs and gaps, we carried out a scoping study, a review method aimed at mapping evidence (e.g., peer-reviewed articles) to convey the breadth and depth of a field [27, 28]. The aims of this scoping study were: [1] to map the intervention research in which children have participated; and [2] to examine children’s meaningful participation in such research. The extensive scholarship in childhood studies, while not necessarily health-focused, offers critical methodological insights that can assist pediatric researchers to design, implement, analyze, and disseminate more rigorous and insightful qualitative research *with* (rather than on) children. These insights from childhood studies include attending to researcher assumptions and biases, to the interpretation and representation of the data, and to attention to voice and the diversity of children’s experiences [29]. Situating our review findings within childhood studies, we identify avenues to increase children’s meaningful participation in intervention research—a step that is critical for promoting interventions that are relevant to and have positive effects on children’s lives.

Our study is unique among reviews of health research with children because it focuses on health interventions, qualitative research, and middle childhood, across topical and geographical areas. Prior reviews have tended, instead, to use a wide age range when defining childhood and have focused specifically on certain topical areas, disease categories (e.g., HPV vaccination, cancer) [18, 30–34], or the ethical and methodological challenges that researchers face when working with children [35]. Further, reviews have not attended to health interventions (e.g., programs, processes, and guidelines aimed at affecting health outcomes), a necessary target for improving the use of evidence produced with children in intervention design, implementation, and evaluation.

## Methods

Our team included four PhD level researchers who work in medical schools and have degrees concentrated in childhood studies as well as in public health and medicine (the team included one MD/PhD and a medical student). We conducted and reported the review according to the Preferred Reporting Items for Systematic Reviews and Meta-Analyses extension for scoping reviews (PRISMA-ScR) guidelines, the five-stage framework outlined by Arksey and O’Malley, and the modified Levac et al.’s scoping study framework [27, 28, 36]. Levac et al.’s framework consists of six stages: identifying the research question; searching for relevant studies; selecting studies for inclusion; charting the data; collating, summarizing, and reporting the results; and consulting stakeholders. We carried out the first five stages and organized our methods section according to these stages. A published protocol does not exist for this scoping review.

### Identifying the research question

The question that guided our study was: What is the state of participation of children ages 6–11 years in health intervention research? To formulate our question, we drew on the PCC (Population, Concept, and Context) elements of designing a scoping study question [37]. Our population of interest was children ages 6 to 11 years. The concept we employed was *health intervention research,* defined broadly to include empirical research on interventions with children, including research on programs, processes, and guideline and including all points on Proctor’s conceptual model of implementation research from implementation strategies to outcomes [38]. In selecting search terms we recognized that the language of participatory research rarely enters the lexicon of intervention science where studies may nonetheless include children as stakeholders. We therefore elected to use broader search terms while focusing on OVID Medline in order to balance a higher tolerance for terminological variation with the time constraints of manually assessing a higher number of articles by the inclusion criteria.

Research *with* children was defined as research in which children are “actively participating and expressing their views and opinions” [39]. We focused only on qualitative studies for this review because of the increasing emphasis on the value of qualitative data for intervention development, adaptation, and evaluation [2, 40, 41]. The context was open, including all geographical, healthcare, and sociocultural contexts.

### Searching for relevant studies

We worked with a medical librarian and conducted a comprehensive search of Ovid Medline for literature on child participation in qualitative health intervention research. The search (completed July 3, 2018) encompassed a decade (January 1, 2007 to July 2, 2018) for breadth. Medline was selected as it is the prominent bibliographic database of life sciences and biomedical information and the most appropriate database to search for health intervention research. The decision to focus solely on Medline was made in conjunction with our medical school’s librarian who has expertise in scoping and systematic reviews. She identified that, because our goal was to map the health intervention research, a search of Medline was most strategic. It would capture the state of the research and also how researchers in the medical and health sciences typically search scholarly work to inform their interventions. The search included the following keywords: “(children) AND (health intervention) AND (participation) AND (qualitative research),” as well as the corresponding Medical Subject Headings terms, restricted to middle childhood (6–11 years) (see Supplementary 1). Grey literature was not searched as we focused on intervention research that is published in peer-reviewed journals to understand the state of the scholarly field.

### Selecting studies for inclusion

To determine study eligibility, the following criteria were applied: children 6–11 years old; published from 2007 to the present (date of search – July 2, 2018); included a health intervention; study designs were qualitative; and studies were published in English. We included studies that had children below age 6 and above age 11 *if* those studies also included children in our target category (6–11 years). We did this because study participant samples often do not map directly on to specific age ranges. We focused only on qualitative studies for this review because there is increasing emphasis on the value of qualitative data for intervention development, adaptation, and evaluation [1, 2]. Studies were excluded if they were unpublished studies, review articles, or not focused on health.

Search results were screened in EndNote X8. The selection of sources of evidence was undertaken by two authors (JH, CM) for both title and abstract screening and full text review. To ensure that we did not miss articles for relevant inclusion criteria, such as a qualitative components as part of a range of methods, we did full text reviews of all articles in which authors did not definitively detail participants or methods in their abstracts. All full texts were read to identify if they fit our definition of intervention research. We defined pre-intervention and/or pre-implementation as any article that made it clear that intervention creation and/or implementation was the next step (eg. this included studies reporting on a needs assessment). We made this decision to limit the ambiguity and author guessing about what types of study could be useful for intervention development or implementation. JH and CM independently reviewed and met to iteratively ensure agreement, and JN resolved any differences in agreement until consensus was reached on all articles.

### Charting the data

Relevant data from the included articles were charted by the research team (JH, CM, JN, and 3 research interns) and recorded in a Microsoft Excel spreadsheet. JH and JN created categories for the chart and refined the categories in an iterative process of testing the data chart. The finalized chart included: author, title, year, research country, authors’ countries, age of youngest child, age of oldest child, adults included in research, family included in research, rationale for including children, aim of paper, when in the implementation process children participated in research (i.e., methods, methodological adaptations, participatory research, meaningful participation, arts-based research, ethical issues discussed, power dynamics discussed), and a range of categories for conditions and social issues covered in the research (e.g., disability, cancer). All team members extracted data and 90 (78%) articles were double extracted with JH and JN resolving disagreement through discussion at weekly meetings. A critical appraisal of individual sources of evidence was not conducted for this review.

### Collating and summarizing the results

Following Arksey and O’Malley [27], we used *descriptive* and *content* analysis as our framework for examining our findings. First, descriptive analysis mapped the studies, focusing on the dissemination of the studies, geographical distribution of studies and researchers, and the types of interventions. Second, we used a combination of descriptive and content analysis [42] to examine age and research methods used. For example, we coded not only for the protocol related to age and method, but also examined how authors discussed age and how/if they reported on findings by age and how they discussed any challenges or benefits of methods used with children.

We coded articles’ rationales for children’s participation to understand why children were included in the research. To minimize subjective inferences, we only coded authors’ explicit statements of why children were included. We documented what methods were used with children and the descriptions and justifications for these methods to understand what constituted research *with* children *for* the researchers.

To examine meaningful participation, we used Roger Hart’s distinction between participation and non-participation [14]. Hart developed a widely-used Ladder of Participation, which has 8 rungs. Rungs 1–3 cover what Hart refers to as non-participation (e.g., token participation, participation only as decoration, and participation manipulated by adults). We use the term “limited participation” to describe studies that fell into rungs 1–3 or for which there was not enough information available to assess participation. We defined meaningful participation as any study that fell in Hart’s category of participation, rungs 4–8. Meaningful participation covers many ways of including children from having them assigned roles by adults to children participating in decisions about the project. We included this wide range of participation within the category of “meaningful” because we recognize that although the ladder metaphor suggests a hierarchy of value, childhood studies scholars have critiqued assumptions that higher rungs of the ladder necessarily produce higher quality or more ethical research [43]. Through discussion of a sub-set of articles and drawing from childhood studies theory, we developed a basic schema for evaluating each study based on Hart’s ladder. Our evaluation schema incorporated considerations of children’s meaningful inclusion at various stages of the research process, including sampling, study design, analysis, reporting, and application of findings. We used only the information available to us from the articles for our assessment and did not reach out to authors for additional information. Because studies were heterogenous and there are many dimensions to children’s meaningful inclusion, we applied the schema flexibly and with consideration for the research goals. Three co-authors trained in childhood studies (JH, JN, JS) coded studies according to the best-matched ladder rung and provided brief justification for the assigned code. A sub-set of articles, including all articles assessed as below level 3 (limited participation), were double coded with high levels of agreement between coders. While coding the articles, each coder kept memos on the article related to the coding schema, and we used these memos, alongside our other findings, to develop a summarizing reflective guide. We note that this guide is not a validated tool but rather a summary of the key issues we identified.

## Results

**Fig. 1:**
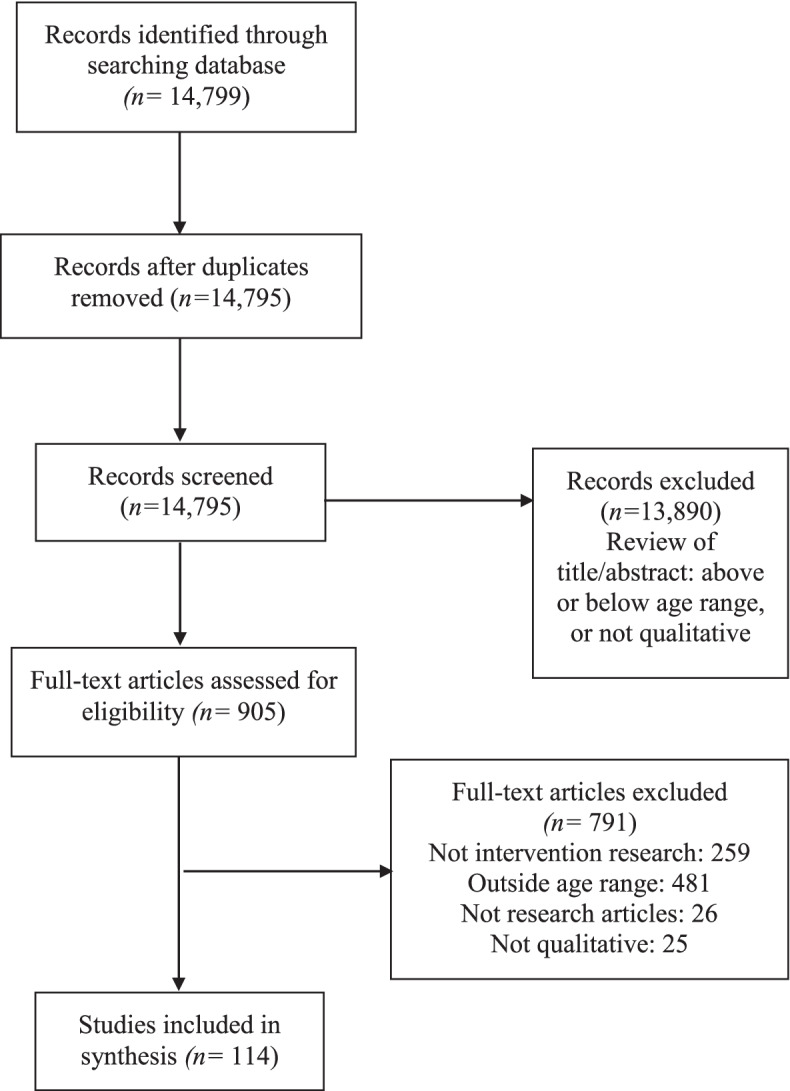
PRISMA-ScR flow diagram showing selection of sources of evidence

The search resulted in 14,795 citations. 13,890 did not meet inclusion criteria based on review of title/abstract alone (e.g., all participants above or below age range, no qualitative methods used, when methods explicitly stated in abstract). We screened 905 full-text papers and excluded 532 based on inclusion criteria and another 259 which were determined to be “not intervention” research (Fig. 1). This resulted in the inclusion of 114 articles (Supplementary Table 3).

### Study characteristics

For an overview of children’s participation in intervention research, we documented what types of health issues were targeted, where the research took place, when children were involved, who participated in the research and how they participated. Most studies targeted disease prevention (*n* = 77, 67.5%), which included physical activity and nutrition interventions, tobacco and other substance use prevention, HPV vaccination, and HIV prevention. Physical activity, nutrition, and obesity prevention interventions were the most prominent, making up 39.5% of the total sample of articles (*n* = 45). Only a quarter of the reviewed studies (*n* = 30, 26.3%) covered illnesses that children actively dealt with in the present (e.g., cancer, cystic fibrosis, diabetes). Even fewer focused on children living with and/or managing the care and health of family members (*n* = 7, 6.1%). Perhaps related to the topical foci, most studies took place in high-income countries, such as the United States (*n* = 41, 36.0%) or the United Kingdom (*n* = 23, 20.2%). Few studies (*n* = 18, 15.8%) were carried out in low- or middle-income countries (e.g., Brazil, India, Zambia).

In assessing the point at which children were included in the research, most studies (*n* = 69, 60.5%) included children during and after intervention development and implementation. Thirty articles (26.3%) reported on research with children prior to an intervention. Out of all articles reviewed, only 15 (13.2%) reported on research with children both before and after implementation.

We identified clear trends in the numerical age and also the age categories (e.g., child, youth, adult) of participants included in the studies. While our inclusion criteria dictated that all studies had participants aged 6–11 years, more studies focused their research with children in the later years of middle childhood (ages 9–11). Further, most studies also included participants outside of our study age range. For example, 84 studies (73.7%) also included children above 11 years. Given that younger and older children were also part of these studies, we assessed the age range of the child participants and identified that 52 (45.6%) studies had a ≥ 5-year difference between the youngest and oldest participants. In addition to children, adults (e.g., teachers, parents) participated in the majority of studies (*n* = 74, 64.9%).

When engaging children in the 6–11 year age group, the most common qualitative methods used were focus groups (*n* = 74; 64.9%) and interviews (*n* = 47, 41.2%), with lesser-used approaches including observational (*n* = 22, 19.3%) and arts-based methods (*n* = 16, 14.0%). Forty-two (36.8%) used more than one qualitative approach (e.g., both focus groups and interviews). We discuss trends in meaningful participation related to age and method in detail below. For more details on study characteristics, see Table 1.

**Table 1:** Peer-reviewed studies involving children’s participation in health interventions (*n* = 114)

Study Characteristics	No. Studies	Total Studies, %
Year of Publication		
2007–2009	17	14.9%
2010–2012	28	24.6%
2013–2015	36	31.6%
2016-July 2, 2018	33	28.9%
Study Locations		
North America	52	45.6%
Europe	35	30.7%
Africa	10	8.8%
Australia and New Zealand	9	7.9%
Asia	5	4.4%
Central and South America	2	1.8%
Multiple continents	1	0.9%
Intervention topics		
Physical activity, nutrition, obesity prevention/ management^a^	45	39.5%
Other, disease prevention and health promotion (e.g., HIV, farm safety)	32	28.1%
Other, management of illness or impairment (e.g., diabetes, asthma)	27	23.7%
Illnesses in family (e.g., parent with cancer)	7	6.1%
Improving care and experience during hospitalization	3	2.6%
Timing of children’s participation		
Only prior to implementation	30	26.3%
Only during and/or after implementation	69	60.5%
Both before and after implementation	15	13.2%
Qualitative methods used		
Focus group	74	64.9%
Interviews	47	41.2%
Observation	22	19.3%
Arts-based	16	14.0%
Free text box on questionnaire	3	2.6%
Other (e.g. network mapping, sharing circle, visual prompts)	7	6.1%
Youngest child participant^**b**^		
Under-6	14	12.3%
6 to 8	37	32.5%
9 to 11	61	53.5%
Oldest child participant		
6 to 8	7	6.1%
9 to 11	21	18.4%
12 to 14	45	39.5%
Over-14	39	34.2%

^**a**^Does not include physical activity or diet aimed at managing effects of disease or disability (e.g., diabetes, cerebral palsy)

^**b**^Two studies used only means to describe participant ages

### Why were children included?

Seventy-four articles (64.9%) included the authors’ rationales for including children. We grouped rationales into two inductively developed categories to classify the authors’ intents. The first category linked their rationales to children as inhabiting a distinct social category and to children’s “unique worldviews and perspective” that had been “dismissed,” “missing,” or “excluded” from previous intervention development and implementation [44–46]. Forty-six articles (40.4% of the total sample; 62.2% of the articles stating a rationale) fit this child-focused category. The second category focused on child participants as “stakeholders,” “target populations,” “consumers,” “patients,” “community members,” and “intervention users,” and included 28 articles (24.6% of the total sample; 37.8% of the articles with rationales). In contrast to the first child-focused category, this stakeholder category made no mention of children’s age category or of childhood as a social category as part of the rationale for including them.

When compared, these two categories had similarities and differences. Both sets of rationales focused on concepts of voice and empowerment as a central reason for including children as participants. This focus on voice and empowerment, however, seemed to draw from different literatures. The latter category that focused on stakeholders and target groups was aligned more with a disease and health focus that places emphasis on learning from those who experience a particular health issue. However, the emphasis in the former child-focused category (i.e., childhood as a social category) was on childhood status as disempowering, and those researchers were more explicit about addressing age-related power dynamics during the research process. That is, the researchers sought to critically consider children’s position in their society in relation not just to the topical focus, but also in relation to the researchers. Most explicitly, this set of articles focused on methodological adaptations to facilitate children’s inclusion by disrupting common ways of interacting between children and adults that might discourage children’s participation or feelings of inclusion. Some adaptations included inviting children to lead group discussions [47], sit at eye level [48], or address adults by their first names [49]. Additionally, this set of articles was more likely to incorporate participant observation [50] and use task- and arts-based methods (e.g., drawing, photography, performance) [51].

### Children’s meaningful and limited participation

Using Hart’s distinction between meaningful child participation and non-participation [14], we assessed children’s participation as meaningful in 83 studies (72.8%). We identified two primary ways that researchers in the meaningful participation category engaged children in intervention research. The first approach was more structured, with researchers asking children directed questions aimed at the adaptation and evaluation of intervention materials. Children participated in content design, assessed the content of messaging, and offered their opinions on what they liked or disliked and why [46, 52–56]. In the second approach, researchers focused more broadly on identifying children’s knowledge and experiences of particular health topics and interventions. Such work was often based on interpretivist paradigms in qualitative research, often with a child-focused rationale and using open-ended interviews, task- and arts-based methods, participant observation, and multiple methods (e.g., see [44, 51, 57–61]). For example, Meininger et al. [62] examined how children of different ages experienced and perceived food and physical activities, and then they drew on their perceptions to tailor an intervention. Bond et al. [51] identified that children in Zambia were wary of an intervention that invited children to promote tuberculosis testing in their households, because of the stigma related to tuberculosis and children’s subordinate positions in relation to adults, but that the children also wanted to be involved. They tailored their intervention to take into account TB stigma, children’s positions in households, and children’s desires to participate. Thus, research with children could directly shape delivery of interventions to be more sensitive to children’s experiences or to understand the uptake of interventions during and after completion. Supplementary Table 2 offers examples of the range in ways that authors meaningfully engaged children.

The 31 (27.2%) remaining articles categorized as limited participation were informative of when and how children’s participation may get tokenized. Many did not include enough information to assess the meaningfulness of children’s participation, and thus we could not classify these articles in the category of meaningful participation. Some common reporting approaches that made it difficult to assess children’s meaningful participation included a lack of information about the methods used with children and the exclusive use of adult and youth (> 11 years) quotes and responses in results and discussion sections, without any mention of the younger children in those sections. Additionally, many of the articles in this category were studies with participants of different ages and roles (e.g., child and parent, student and teacher). Articles in this limited participation category also conflated child and adult responses, using statements such as, “adults and children said,” making it difficult to ascertain how much children informed findings or conclusions.

Assessing the content of the articles in the limited participation category brought to light limitations we were also identifying in articles in the meaningful participation category. Notably, we also identified this “adults and children” conflation in reporting results in articles in the meaningful participation category, albeit to a lesser extent. To offer a further example, we identified a strong trend within the meaningful participation category to *not* report or discuss younger children’s quotes or other data (e.g., drawings and their explanations). As reported above, numerous studies included participants above the age of 6–11. The perspectives of older children (i.e., close to and above 11 years) and adults were more often reported than those of the children in our scoping review’s age range. Some researchers reported *why* they focused more on older age ranges, often identifying methodological challenges in working with younger children in their samples. For example, Laroche et al. [63] began over-sampling children above age 12 during recruitment because, in their perspective, they “express [ed] themselves more clearly than younger children” (p. 428).

The omissions of children’s quotes and other data from results and discussion sections of studies in our review, even those with meaningful participation, might suggest that many researchers also found difficulties including and interpreting the perspectives of children in middle childhood. Hieftje et al. [44] noted that children in their study produced unanticipated and difficult to interpret responses. However, they argued against disregarding such responses: “Although researchers might intend for an activity to open discussion about a particular topic, young adolescents may use the activity in a different way … The data that subsequently emerge … provide poignant insights into young adolescents’ beliefs, attitudes, and behaviors” (p. 720). This suggests the need to further efforts and upskill researchers in how to not just carry out methods with children, but also analyze the data produced by children (e.g., quotes, stories, drawings, performances, and other artwork). Finally, we note in Table 1 an increase in annual number of publications over the review time period (2007-July 2, 2018). However, we did not identify an increase in meaningful participation through this time period, which further supports the need for upskilling.

## Discussion

We carried out this scoping study to describe the state of research on the participation of children in health intervention studies. To examine trends and gaps, our study was purposefully broad, rather than disease- or intervention-specific or focused on a particular implementation time point (e.g., pre-implementation; post-implementation). Our review showed that the participation of children in intervention research is still in its nascence, with just 114 articles meeting a broad inclusion criteria. Although we did note an increase in the annual number of publications involving children’s participation over time, this increase could be due to a number of factors other than an increase in research with children (e.g., more general publishing trends). Importantly, *meaningful* participation numbers did *not* increase with time, suggesting that the time has come for more purposeful and strategic approaches to including children in middle childhood as active research participants in intervention planning, implementation, and evaluation.

**Fig. 2:**
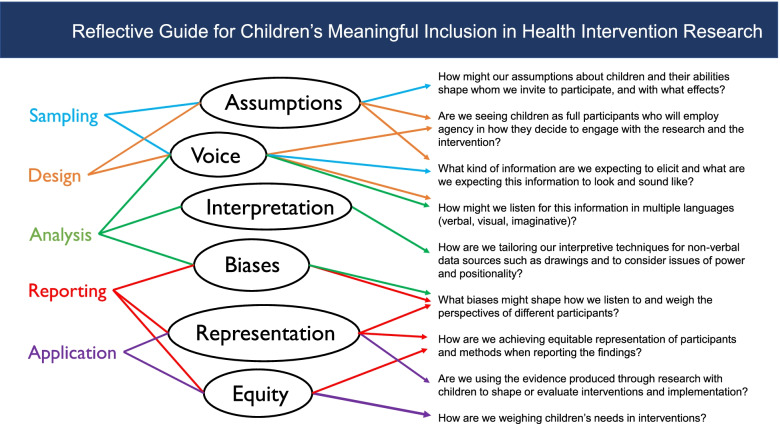
Reflective guide for children’s meaningful inclusion in health intervention research

Based on the scoping review findings and drawing on the well-defined research on children as research participants in childhood studies, we identify three areas for moving the field forward to include children’s perspectives and experiences more fully into child health interventions. These three areas are recognizing and addressing trends and gaps, improving methodological clarity and diversifying methodological approaches, and attending to power structures that hinder meaningful participation of children in research. We summarize these in a reflective guide that draws from our findings (Fig. 2), and which may assist researchers who wish to deepen the meaningfulness of children’s participation at all stages of health intervention research (Fig. 2).

### Recognizing and addressing trends and gaps

The review revealed clear patterns in the time point at which children were engaged in the research on interventions as well as in topical and geographical areas. First, most articles did not seek children’s input early on in the research/intervention development process; at best they engaged children during or immediately prior to implementation. As we showed above, some researchers who engaged children in pre-implementation research gained valuable input from children as to how to adapt their interventions to children’s lives and to children’s strengths and needs [43]. Without this initial involvement, researchers miss an opportunity to improve interventions and their implementation. They may in fact introduce interventions that do not address children’s needs or that could cause harm to children.

Second, there was a strong trend in the focus of interventions, with most focusing on disease prevention, especially on physical activity and nutrition interventions. While this focus is important given the range of health issues throughout the lifespan and the link between childhood obesity and obesity in adulthood [64, 65], a critical perspective from childhood studies offers a different insight on this topical trend. Childhood researchers in the social sciences have identified a tendency for researchers to view children in terms of their futures as adults, often focusing on future needs, health, and productivity [66]. They have shown that viewing children as “future adults” can limit researchers’ understandings of children in the present and can miss out on issues, including health issues, that children find meaningful and that actively affect children now [67, 68]. Our review raises the question: Are such interventions more common because the adults funding and designing studies are viewing children in future terms? What effects might such views have on the interventions? What types of health issues might children focus on and what are we missing out on related to children’s illness experience during childhood?

Third and relatedly, we note that most studies reported on research with children living in high-income countries. As such, we are missing important perspectives from children on the topics covered in the review that affect children in both high- and low-income countries and on specific health issues that children may face in low-income countries (e.g., tuberculosis, waterborne illnesses). This is likely indicative of global inequities in academia, where researchers working in high-income countries have greater access to research funding to carry out work, and to the institutional and social resources to publish such research in the journals indexed by Ovid Medline. However, given that childhood studies is based on the premise that childhood is not singular, but experienced in context, developing an understanding of children’s involvement in intervention research will be at best partial if it is only done with certain populations living in certain contexts [68, 69]. Further, as global health research and interventions increase, we would warn researchers, specifically those from high-income countries who work in low-income countries, that mapping understandings of childhood in one place onto those in another could lead to ineffective or detrimental interventions [70].

### Methodological clarity and diversity - moving qualitative research forward to support children’s participation

Several gaps we identified relate to a need to improve methods and the interpretation of findings within qualitative approaches to intervention research. First, many articles lacked sufficient clarity about methodological choices and adaptation, the process of engaging children in the qualitative research, and analytic approaches, as Larsson et al.’s review of young people’s (ages ≤25 years) participation in intervention also identified [18]. This was especially the case in the studies that included adults. Reporting details about research with children may be a challenge for authors who face publisher word and image restrictions, or who need to describe multiple methods and participants. Researchers may also face constraints related to publishing in more traditionally quantitative journals because of much lower word counts or the need to revise articles based on comments from peer reviewers unfamiliar with qualitative research, who may de-emphasize the qualitative methods components or need standard qualitative terms defined in greater depth. Nevertheless, the lack of clarity and detail made it difficult to assess the rigor of the qualitative work and to understand how children participated in projects or if their participation was meaningful.

Second, our review identified a reliance on two types of qualitative methods in published research on intervention work— focus groups and interviews. While both provide useful insights and are standard in qualitative research, they are not the only options available, and they may not always be the best approaches for answering certain research questions, or for working with children in middle childhood [71]. Childhood studies offers a wealth of techniques and approaches for working with children, from ethnography to more visual and arts-based approaches, that can yield valuable insights for health interventions [3, 7, 15, 16, 72]. In sum, investment in qualitative approaches is likely to enhance implementation of interventions, as extending the range and improving the rigor of qualitative techniques in health intervention research will enable meaningful interpretations of children’s data, secure trust in those interpretations, and promote the value of research with children.

### Attending to power structures - toward more meaningful participation of children in intervention research

Deepening qualitative approaches and qualitative reporting is not enough to facilitate children’s meaningful participation without an understanding of both children’s and researchers’ positions and voice in research. Our analysis of rationales for inclusion of certain populations can offer insight into researchers’ theoretical framings, assumptions, and ethical approaches, especially in empirical articles on interventions, where word counts and conventions may constrain authors from detailed discussion. Childhood studies researchers have demonstrated the importance of attending to children’s social positions (e.g., as structurally dependent, bound by different institutional and social rules) in order to promote their inclusion [73]. Without a consideration of power relations that lead to children’s exclusions in research, researchers might use methods and promote interpretations that reinforce relations of power (e.g., teacher, student) and dampen children’s voices (e.g., conducting a study as part of a class activity where children may not be able to decline participation). Asking children to participate without attending to the power dynamics that shape interventions may in fact be disempowering to young people (e.g., when researchers tell children that they will listen to their input but do not reveal their own limits to make the children’s suggested changes). The studies in our review in which authors gave explicit attention to power inequities between children and adults seemed to find points of meaningful inclusion through methodological adaptations (e.g., using drawing or photography) and in tailoring of interventions to children’s needs.

Relatedly, our review identified substantial gaps in the representation of children’s participation in research reporting. Some researchers expressed legitimate uncertainties around how to interpret and represent children’s responses in order to apply these responses to interventions. And many articles exhibited biases in how they weighed and reported on younger children’s versus older children’s, youth’s, or adults’ responses. These gaps in representation in our scoping study do not speak to the impossibility of including younger children; childhood studies researchers have amassed substantial evidence demonstrating the inclusion of younger children. It speaks, instead, to the need to broaden the concept of what child participation looks like and how to represent children’s perspectives and experiences [6, 10, 74]. The challenge, as we see it, is in how to represent children’s responses as some among many, without diminishing or tokenizing them or over-selling children's agency in relation to those in more powerful positions [23].

#### Summarizing findings into a reflective guide

To support this work toward children’s meaningful participation, we have designed a reflective guide to summarize our findings (Fig. 2). The guide examines the various stages of the research process that our review delineated: sampling, design of intervention and/or of implementation process, analysis of the data, reporting of the data, and application of findings. We developed this guide by distilling six main challenges or child-centered ‘lenses’ (e.g., representation, voice, interpretation, biases, representation, equity) from the themes and gaps we identified in this review. Lines guide the reader from each stage of the research process through key lenses and refract toward questions designed to help researchers reflect on relevant aspects of children’s inclusion. This guide is not intended to be an exhaustive recipe for achieving children’s meaningful participation, rather, a summary of findings and a resource for establishing routine practices of child-centered thinking that create the foundations of a participation ecosystem for children’s inclusion in health intervention research.

#### Limitations

Our goal was to identify the breadth of children’s participation in health intervention research. To do so, we searched Ovid Medline and adopted a broad definition of intervention research. This approach was reasonable given our intention to scope the state of the field for health researchers carrying out health-related intervention and implementation work. We made this decision with the medical librarian to identify the breadth of how children were engaged in more-medically-oriented intervention research, without limiting our search to a too-narrow definition of their participation or of interventions. On just Ovid Medline, this approach returned 14,799 articles and, thus, adding other databases would have made the project unfeasible. The scoping review methodology, while systematic, is flexible to account for such decisions. Our analysis was limited to information provided in the articles, and to a specific time frame. While this may not offer a full picture of children’s involvement, it offers important insight into trends in children’s participation, as well as in publishing and reporting gaps. Trends in research change, and we anticipate that an updated search may find changing trends in topical focus, such as the inclusion of children in research on interventions to manage, for example, COVID-19 or on the implementation of digital tools, such as eHealth, to be used by children and their providers (e.g., [75]). However, while topics may come in and out of fashion, our review shows that there is much work to be done to shift research, publishing, and reporting shortcomings that result from publishing norms, entrenched adultist modes of thinking and researching, and the need for deeper engagement with theory from childhood studies at all stages of the research process.

## Conclusions

While our review shows that children have a place in intervention research, we identified several trends and gaps that need addressing. It is telling that of the nearly 15 thousand articles initially identified via our search only 114 could be included in this scoping review. Obviously, there is much to be done to increase the quantity of qualitative research focusing on children in middle childhood. The subsequent points focus on improving the quality of that work. First, the topical and geographical focuses of the work were limited, and thus offer a skewed view of childhood, the health problems children contend with, and the types of interventions that are appropriate. Second, we demonstrate the need to parse how meaningful participation diminishes with age and the importance of focusing on younger age groups (in contrast with adolescents and adults) to ensure that participation is equitably promoted. Finally, we identified key areas for upskilling health scientists in qualitative paradigms and childhood theory and methods. Greater interdisciplinarity between childhood studies and health fields could help to strengthen researchers’ critical skills toward more purposeful and effective interventions. Addressing these gaps might improve the impact that research *with* children will have on interventions and, we would argue, the returns that such interventions might bring to child, family and population health.

## Supplementary Information

**Additional file 1.** 

**Additional file 2.**

**Additional file 3.** 

## Data Availability

All data generated or analysed during this study are included in this published article [and its supplementary information files].
