# Supplementary material for: What is the state of children’s participation in qualitative research on health interventions?: a scoping study
Source: BMC Pediatr. 2022 Jun 4;22:328. doi: 10.1186/s12887-022-03391-2 (PMC9166159; doi:10.1186/s12887-022-03391-2)
Supplement: Supplementary file 1 — Additional file 1. [file 12887_2022_3391_MOESM1_ESM.docx]

**Supplement 1: Ovid Medline Search**
**Date limit: January 1, 2007-July 2, 2018
14,799 results**

“Children AND health intervention AND ethnography” OR “Children AND health intervention AND focus groups/participation AND qualitative research”

**Children**
exp Child/ or exp Child, Preschool/ or exp Adolescent/ OR (Young adj1 (person* OR people)).ti. OR (Young adj1 student*).ti. OR Child.ti. OR Children*.ti. or Adolescent*.ti. or Teen*.ti. or Youth*.ti. or Adolescence.ti. or girl*.ti. or boy*.ti. or juvenile*.ti. or schoolboy*.ti. or schoolchild*.ti. or schoolgirl*.ti.

**Focus groups/participation**(focus adj1 group*).mp. OR Exp focus group/ OR collage.mp. OR skit.mp. OR drawing*.mp. OR (role adj1 play*).mp. OR storytell*.mp. OR photo mapping.mp. OR photomap*.mp. OR narration.mp. OR participant observation.mp. OR child friendly.mp. OR child oriented.mp. OR research with children.mp. OR ((interview* OR participat* OR interact* OR agent* OR consume*) adj3 (youth OR child or children or adolescent* or kid* OR schoolchild* OR schoolboy* OR schoolgirl* OR teen*)).mp. OR feedback.mp. OR mosaic.mp. OR "train the trainer".mp. or journaling.mp. OR photovoice.mp. OR Exp community-based participatory research/ OR ((questionnaire* OR survey*) adj25 (youth OR child or children or adolescent* or kid* OR schoolchild* OR schoolboy* OR schoolgirl* OR teen* OR student*)).mp. OR Exp "Surveys and Questionnaires"/

**Health Intervention**
((Health.mp. or wellness.mp. OR HIV.ti. OR cancer.ti. OR tuberculosis.ti. OR Exp "bacterial infections and mycoses"/ OR Exp cardiovascular diseases/ OR Exp chemically-induced disorders/ OR exp "congenital, hereditary, and neonatal diseases and abnormalities"/ OR Exp digestive system diseases/ OR Exp disorders of environmental origin/ OR Exp endocrine system diseases/ OR Exp eye diseases/ OR Exp "female urogenital diseases and pregnancy complications"/ OR Exp "hemic and lymphatic diseases"/ OR Exp "immune system diseases"/ OR Exp "male urogenital diseases"/ OR Exp "musculoskeletal diseases"/ OR Exp neoplasms/ OR Exp Nervous system diseases/ OR Exp "nutritional and metabolic diseases"/ OR Exp occupational diseases/ OR Exp otorhinolaryngologic diseases/ OR Exp parasitic diseases/ OR Exp respiratory tract diseases/ OR Exp "skin and connective tissue diseases"/ OR Exp stomatognathic diseases/ OR Exp virus diseases/ OR Exp "wounds and injuries"/) AND ("service delivery".mp. OR Intervention*.mp. OR program*.mp. or strategy.mp.)) OR Exp health promotion/ OR Exp health education/ or "health education".mp. OR exp risk reduction behavior/ OR exp risk assessment/ OR exp health services research/ OR prevention & control.fs.

**Ethnography**ethnograph*.mp. OR participant-observation.mp. OR participant observation.mp.

**Qualitative research**Exp qualitative research/ OR qualitative research.mp. OR exp attitude to health/ OR attitude*.mp. OR Exp health behavior/ OR (health adj1 behavior*).mp. OR narrative.mp. OR Exp decision making/ OR Exp healthcare disparities/ OR (decision* adj1 mak*).mp. or decision-making.mp. OR psychosocial.mp.
