# Supplementary material for: What is the state of children’s participation in qualitative research on health interventions?: a scoping study
Source: BMC Pediatr. 2022 Jun 4;22:328. doi: 10.1186/s12887-022-03391-2 (PMC9166159; doi:10.1186/s12887-022-03391-2)
Supplement: Supplementary file 2 — Additional file 2. [file 12887_2022_3391_MOESM2_ESM.docx]

| **Supplementary table 2: Examples of meaningful participation as extracted from scoping study*** | | | | |
| --- | --- | --- | --- | --- |
| Meaningful Participation | | | | |
| **Citation** | **Study Focus** | **Description of child participation** | | |
| Block et al. 2012 | "To evaluate a structured cooking and gardening program in Australian primary schools, focusing on program impacts on the social and learning environment of the school." | Authors sought children's understandings of and experiences with a garden program to identify the value of the program to children. Children frequently quoted and authors contextualized their interpretation of quotes. | | |
| Bryant & Hoon 2007 | "To examine how rural students read farm safety messages in printed farm safety communication mediums." | Children invited to comment on farm safety media communication tools. Made comparisons between age groups to identify need for age-related communication strategies. Detailed contextualizing of approach to age differences in terms of what this means for farm safety. Extensive reporting of children's responses and how these were interpreted. | | |
| Gadin et al. 2009 | "To analyse if young students could be substantive participants in a health promoting school project." | Children invited to be active in prioritizing change and defining problems, as well as in the change process. "Following classroom-based participatory discussions, children were asked to suggest proposals for what they want changed in their school in order to enhance their health. Children were involved in evaluating, prioritizing, and implementing suggested changes." | | |
| Hampshire and Matthijsse 2010 | "To present a theoretically-grounded analysis of the transformative potential of ‘SingUp’, a £40 million UK government-funded singing initiative for primary school-aged children." | Carried out 18 months of fieldwork with the young people to examine young people's experiences with the intervention through time and "using a range of anthropological methods, underpinned by participant observation." Researchers also used sociological theories about forms of capital to interpret their child-centered data. | | |
| Hieftje et al. 2014 | "To seek information and ideas from the priority audience that would help us create authentic story lines and character development in the video game." | Adolescents' storytelling and photography provided insight into their lives and goals to serve as basis for videogame. The creators consulted adolescents to ensure that the game resonated with them. | | |
| Stinson et al. 2012 | "To explore information needs of children...and their parents in order to develop a web-based psychoeducational program aimed at improving their quality of life." | Invited children and parents to discuss, as dyads, their needs for a web-based tool to help manage arthritis. Assessed the interdependence between children and parents and the ways in which children's and parents' needs differed. Researchers did not overly privilege parent perspectives at the expense of the children's perspectives, and they included a number of quotes from children at the younger end of the age range. | | |
|  |  |  |  |  |
| * Sample derived to illustrate the range of ways research could represent meaningful participation. | | | | |
