# Supplementary material for: What is the state of children’s participation in qualitative research on health interventions?: a scoping study
Source: BMC Pediatr. 2022 Jun 4;22:328. doi: 10.1186/s12887-022-03391-2 (PMC9166159; doi:10.1186/s12887-022-03391-2)
Supplement: Supplementary file 3 — Additional file 3. [file 12887_2022_3391_MOESM3_ESM.docx]

| **Supplementary table 3: Studies included in the scoping review with select data extracted** | | | | | | | |
| --- | --- | --- | --- | --- | --- | --- | --- |
| **Citation** | **Study aim** | **Country of study** | **Ages of child participants** | **Qualitative methods with children** | **Study rationale linked to childhood as a social category** | **Adults included in research** | **Timing of children’s participation** |
| Aarons et al. 2010 | “To examine the degree to which youths and caregivers attend to different factors in evaluating their experiences with mental health programs.” | USA | 11-17 | Interview | Yes | Yes | During implementation |
| Amaya-Castellanos et al. 2015 | “To provide a step-by-step description of the design and implementation of an educational intervention to promote healthy eating and physical activity called ‘Healthy Recess.’” | Mexico | 8-12 | Open-ended questions on a questionnaire, observation of intervention workshops | No | Yes | Pre-implementation |
| Arora et al. 2013 | “To explore contextual influences that enable initiation of different forms of tobacco use among youth...and their perceptions about tobacco cessation.” | India | 10-19 | Focus groups | Yes | Yes | Pre-implementation |
| Arora et al. 2010 | “To evaluate the efficacy of a multi-component community-based intervention model...for the prevention of uptake and cessation of tobacco use among adolescents from low SES, using quantitative research methods.” | India | 10-19 | Focus groups | No | Yes | Both phases |
| Azevedo et al. 2013 | “To identify facilitators and barriers to participation... in the treatment arm of... [a] culturally tailored obesity prevention trial.” | USA | mean age = 8.29 | Interview | No | Yes | During implementation |
| Barnes 2014 | To “examine some social and psychological well-being impacts of extended collaborations between a theatre company and children with communica difficulties.” | United Kingdom | 6-7 | Observations, children’s commentary on a video of the intervention | Yes | Yes | During implementation |
| Barrett et al. 2017 | “To understand beverage choice motivations and test promotional concepts that can encourage Central American Latino urban youth to drink more water.” | USA | 6-18 | Focus groups | Yes | No | Pre-implementation |
| Bekker et al. 2017 | “To investigate students’ tuck shop buying behaviour, choices of lunchbox items and healthy eating perceptions and attitudes at a school with a nutritionally regulated tuck shop and a school with a conventional tuck shop.” | South Africa | 7-14 | Focus groups | No | No | During implementation |
| Bingham et al. 2009 | “To discuss implications for communication strategies to facilitate vaccine acceptance.” | India, Peru, Uganda, Vietnam | 9-16 | Interviews; focus groups | No | Yes | Pre-implementation |
| Blanson et al. 2013 | “Assess the effects of personalised robot behaviours on the enjoyment and motivation of children (8–12) with diabetes, and on their acquisition of health knowledge, in educational play.” | Netherlands | 8-12 | Video observation | No | No | During implementation |
| Block et al. 2012 | “To evaluate a structured cooking and gardening program in Australian primary schools, focusing on program impacts on the social and learning environment of the school.” | Australia | 8-12 | Interviews, focus groups, participant observation | No | Yes | During implementation |
| Boddy et al. 2012 | “To gather qualitative focus group and interview data regarding healthy eating particularly in relation to enabling and influencing factors, barriers and knowledge in children and adults (parents and teachers) from schools within the CHANGE! programme to provide population specific evidence to inform the subsequent intervention design.” | United Kingdom | 9-10 | Focus groups | No | Yes | Pre-implementation |
| Bond et al. 2010 | “To evaluate information dissemination by children and attitudes among children towards a schoolbased tuberculosis (TB) reduction strategy that asked children to address TB symptoms, testing and stigma in their homes.” | Zambia | 11-18 | Focus groups, role-play, drawing | Yes | No | Both phases |
| Bonell et al. 2010 | “Interventions to improve school ethos can reduce substance use but ‘‘upstream’’ causal pathways relating to implementation and school-level changes are uncertain. We use qualitative and quantitative data from a pilot trial to build hypotheses regarding these.” | United Kingdom | 11-12 | Interviews | No | Yes | During implementation |
| Breland-Noble et al. 2010 | “To describe African American adolescents’ experience of depression and suggest mechanisms for improving African American youth treatment engagement.” | USA | 11-17 | Interviews, focus groups | Yes | No | Pre-implementation |
| Bresee et al. 2016 | “To assess the potential for children to be change agents in five schools in rural Zambia.” | Zambia | 8-12 | Focus groups, role-play, drawings | No | Yes | Both phases |
| Bryant & Hoon 2007 | “To examine how rural students read farm safety messages in printed farm safety communication mediums.” | Australia | 7-12 | Focus groups | Yes | No | During implementation |
| Bugge et al. 2008 | “To assess a preventive support program for children aged between 5 and 18 years and their families when a mother or father has an incurable form of cancer.” | Norway | 5-18 | Interviews, drawing, social network map drawings | Yes | Yes | During implementation |
| Cai et al. 2017 | “To develop an acceptable and feasible self-management intervention that addresses the self-identified needs of children and young people with Type 1 diabetes and their parents.” | England | 8-16 | Interviews; focus groups | Yes | Yes | Pre-implementation, During implementation |
| Caleffi et al. 2016 | “Analyze how therapeutic play structured in a nursing care model contributes to the care of hospitalized children.” | Brazil | 5-8 | Participant observation, interviews | Yes | Yes | During implementation |
| Canavera et al. 2008 | “To develop and pilot test a social cognitive theory-based intervention for children to prevent childhood obesity.” | USA | 10-12 | Focus groups | No | Yes | Pre-implementation |
| Clarke et al. 2015 | “To explore parent and child experiences of the WAVES study obesity prevention intervention, in order to gain understanding of the mechanisms by which the intervention results in behaviour change, and provide context to support interpretation of the main trial results.” | United Kingdom | 6-7 | Focus groups | Yes | Yes | During implementation |
| Cottrell et al. 2010 | “To identify community health beliefs and the development of theoretically based materials to increase participation.” | USA | 10-11 | Interviews, focus groups | Yes | Yes | Pre-implementation |
| Dalma et al. 2016 | To “qualitatively identify the perceptions of both parents and students towards healthy eating and related barriers and their experience of a school feeding programme.” | Greece | 9-15 | Focus groups | No | Yes | During implementation |
| Dariotis et al. 2016 | “To explore youths’ own perspectives on stress, stressors in youths’ lives, and perceived changes in responses to stress post-intervention.” | USA | 10-13 | Focus groups | Yes | No | During implementation |
| Davies et al. 2012 | “To explore the feasibility and appropriateness of the 10,000 Steps program resources in the primary school environment.” | Australia | 10-12 | Focus groups | No | Yes | During implementation |
| D’Cruz et al. 2015 | “To explore parent and youth perspectives on a proposed intergenerational game designed to increase effective parent–youth sexual health communication and skills training.” | USA | 11-14 | Focus groups | No | Yes | Pre-implementation |
| Dowd et al. 2015 | “To explore the experience of being in the Go Girls! program from the perspective of its participants.” | Canada | 11-14 | Interviews | No | No | During implementation |
| Dudovitz et al. 2016 | “To describe whether and how corrective lenses affect academic achievement and health.” | USA | 5-14 | Focus groups | No | Yes | During implementation |
| Edgington et al. 2016 | “To assess the feasibility of a new 8-week CBT-based group intervention for self-regulation of sensory processing difficulties.” | UK | 11-16 | Interviews | Yes | Yes | During implementation |
| Eisen et al. 2008 | “To investigate what type of art image children prefer, and what type of art image has potentially stress-reducing effects on children in hospitals.” | USA | 5-17 | Focus groups | Yes | No | Pre-implementation |
| Ejike et al. 2017 | “To test and report “a newly developed health educational game Schisto and Ladders™ and its potential to promote behavioural changes for schistosomiasis control by increasing knowledge of schistosomiasis among school children in an endemic community near Abeokuta, Nigeria.” | Nigeria | 5-19 | Focus groups | No | No | During implementation |
| Ensaff et al. 2015 | “To explore the impact of a school-based kitchen project at a large inner London school.” | UK | 7-9 | Focus groups, with drawing | No | Yes | During implementation |
| Esse et al. 2017 | “To develop an educational cartoon that might help improve school children’s awareness regarding STHs and diarrheal diseases, and related hygiene practices in Côte d’Ivoire.” | Ivory Coast | 9-14 | Focus groups, observation | No | No | Pre-implementation |
| Fernandes Davies et al. 2016 | “To investigate whether AVGs with well-constructed stories elicit desirable cognitive, affective, and behavioral consequences.” | USA | 8-11 | Interviews | No | No | During implementation |
| Gadin et al. 2009 | “To analyse if young students could be substantive participants in a healthpromoting school project.” | Sweden | 7-12 | Participatory discussions, content analysis of children’s proposals for change | No | Yes | Both phases |
| Gillard et al. 2011 | “To examine outcomes associated with participation in a camp for youth with HIV/AIDS.” | USA | 7-16 | Interviews, focus groups, observation | Yes | Yes | During implementation |
| Gilmore et al. 2010 | “To explore the experiences of children participating in modified constraint-induced movement therapy (CIMT) within a circus-themed day camp.” | Australia | 5-16 | Interviews, observation | Yes | No | During implementation |
| Gladstone et al. 2014 | “To understand whether children shared program goals predetermined by adults, and how, or if, the intervention was responsive to their needs.” | Canada | 7-13 | Interviews (individual and group), participant observation, drawing | Yes | No | During implementation |
| Grassi et al. 2016 | “To investigate the impact of a school-based nutrition and media education intervention on the promotion of fruit and vegetable consumption to help prevent childhood obesity.” | Italy | 10-11 | Focus groups | No | Yes | During implementation |
| Grier et al. 2015 | “To evaluate the feasibility (i.e. demand, acceptability, implementation and limited-effectiveness testing) of a 10-week experiential theory-based gardening and nutrition education programme targeting youth living in public housing.” | USA | 5-17 | Interviews | No | Yes | During implementation |
| Guerrero et al. 2016 | “To qualitatively explore the experiences of children who had participated in a 4-week imagery intervention designed to increase active play.” | Canada | 9-10 | Focus groups | Yes | No | During implementation |
| Haerens et al. 2009 | “To describe important changeable influencing factors for dietary behaviors among young children in order to determine the best approaches for developing and implementing a standardized intervention that is feasible for each of the intervention contexts and populations”. | Belgium, Italy, Sweden, Spain, Germany, Hungary, Cyprus, Estonia | 2-8 | Focus groups | No | Yes | Pre-implementation |
| Haerens et al. 2010 | “To describe [various] influencing factors for physical activity among young children to determine the best approaches for developing the IDEFICS community based intervention.” | Belgium, Italy, Sweden, Spain, Germany, Hungary, Cyprus, Estonia | 6-8 | Focus groups | No | Yes | Pre-implementation |
| Haines et al. 2008 | “To assess children’s opinions about participating in a school-based theater program (Very Important Kids) and to determine their perceptions as to how their participation influenced their weight related attitudes and behaviors.” | USA | 9-12 | Focus groups | Yes | No | During implementation |
| Hampshire and Matthijsse 2010 | “To see if the programme, “Sing up”, favorably affects children’s social and emotional wellbeing.” | United Kingdom | 9-11 | Interviews, focus groups, observation | Yes | Yes | During implementation |
| Hansen et al. 2017 | “To examine acceptability, facilitators, and barriers of HPV vaccination visits at SBHCs from the perspectives of adolescents and parents.” | USA | 11-18 | Interviews | No | Yes | Pre-implementation |
| Hieftje et al. 2012 | “To inform the development of an interactive videogame focused on behavior change to reduce risk and promote human immunodeficiency virus (HIV) prevention in young minority adolescents.” | USA | 10-15 | Interviews, focus groups | No | No | Pre-implementation |
| Hieftje et al. 2014 | “To seek information and ideas from the priority audience that would help us create authentic story lines and character development in the video game.” | USA | 10-15 | Interviews, focus groups, storytelling, illustration, photography | Yes | No | Pre-implementation |
| Hind et al. 2014 | “To determine effective schoolbased exercise programs.” | United Kingdom | 7-8 | Focus groups, observation | No | Yes | During implementation |
| Huby et al. 2017 | “To develop and evaluate a web-based information and support application for parents managing their child’s chronic kidney disease.” | United Kingdom | 5-17 | Interviews, draw and tell method | Yes | No | Pre-implementation |
| Hughes et al. 2015 | “To report the focus group perspectives of preteens’ and parents’ experiences with a feasibility intervention entitled PREP-T1.” | USA | 9-12 | Focus groups, participant observation | No | Yes | During implementation |
| Hunsberger et al. 2015 | “To investigate the impact of point-of-purchase calorie information at one rural middle school.” | USA | 11-15 | Interviews | No | No | During implementation |
| Huys et al. 2017 | “To gain insight in implementation practices of school gardens and in perceptions of key members and children towards a school garden.” | Belgium | 10-13 | Focus groups | No | Yes | During implementation |
| Ison et al. 2010 | “To evaluate a disability awareness programme for students aged 9–11 in Australia.” | Australia | 9-11 | Focus groups | No | No | Both phases |
| Kaponda et al. 2007 | “To describe a four-step process used to develop a culturally and developmentally appropriate adolescent HIV prevention program for communities in rural Malawi.” | Malawi | 10-19 | Focus groups | No | Yes | Both phases |
| Katz et al. 2014 | “To describe the development and initial feedback about an HPV vaccine comic book for young adolescents.” | USA | 9-14 | Interviews | No | Yes | During implementation |
| Kools et al. 2008 | “To describe the disease understandings and perceptions related to dietary adherence in children and adolescents with PHL AND to test a dietary intervention, including nutritional supplements and dietary counseling.” | USA | 9-20 | Focus groups (that included some drawing with younger children) | Yes | No | Pre-implementation |
| Laroche et al. 2008 | “To explore how these adults approached providing food for their children and how their children reacted to dietary changes in the household.” | USA | 10-17 | Interviews | Yes | Yes | During implementation |
| Li et al. 2016 | “To investigate how one typical weight loss camp operates and to explore the experiences and perceptions of children attending the camp.” | China | 7-18 | Interviews, participant observation | Yes | No | During implementation |
| Lloyd and Wyatt 2014 | “To evaluate the Healthy Lifestyles Program and whether or not it creates supportive school and home environments for healthy behaviours.” | United Kingdom | 9-10 | Focus groups | No | Yes | During implementation |
| Lloyd et al. 2011 | “To describe the development of the Healthy Lifestyles Programme (HeLP), a school-based intervention to prevent obesity in children, through the first 4 steps of the Intervention Mapping protocol (IM).” | United Kingdom | 9-10 | Focus groups | No | Yes | During implementation |
| Lowes et al. 2015 | “To explore the experiences of children and adolescents with T1D, and those of their carers, concerning living with and managing diabetes, and attending pediatric diabetes services in the UK.” | Wales | 7-15 | Open-ended free text boxes on questionnaires | No | Yes | During implementation |
| MacLellan et al. 2010 | To explore “parent and student perceptions of barriers and facilitating factors influencing the implementation of school nutrition policies.” | Canada | 9-12 | Focus groups | No | Yes | During implementation |
| MacPhail et al. 2013 | “To assess the potential for using the HPV vaccine in the South African public health care system as an opportunity for integrated health care services for adolescents.” | South Africa | 9-14 | Interviews, focus groups | Yes | Yes | Pre-implementation |
| Marciel et al. 2010 | “To develop and assess a web-enabled cell phone, CFFONE™, designed to provide CF information and social support to improve adherence in adolescents with CF.” | USA | 11-18 | Interviews | No | Yes | Both phases |
| Marsac et al. 2012 | “To examine the acceptability and feasibility of child and parent use of The Cellie Cancer Coping Kit.” | USA | 6-12 | Interviews | No | Yes | Both phases |
| Maticka-Tyndale et al. 2007 | To examine “the impact of a primary school HIV education initiative on the knowledge, self-efficacy and sexual and condom use activities of upper primary-school pupils in Kenya.” | Kenya | 11-17 | Focus groups | Yes | Yes | Both phases |
| McGee et al. 2017 | “To explore the nutrition and physical activity perceptions of children for planning a healthy weight curriculum to address childhood obesity in African-American children livingin the Lower Mississippi Delta (LMD).” | USA | 8-13 | Focus groups | Yes | No | Pre-implementation |
| Medeiros et al. 2016 | “To evaluate the process of program implementation in three Brazilian cities among middle school students between 6^th^ and 9^th^ grade (11 to 14 years old).” | Brazil | 11-14 | Focus groups | No | Yes | During implementation |
| Meininger et al. 2010 | “To elicit, from children in kindergarten(K) through sixth grade, perceptions of foods and activities that would inform the design of developmentally appropriate interventions to prevent and reduce childhood obesity.” | USA | 6-12 | Focus groups, with visual prompts | Yes | No | Pre-implementation |
| Mitchell-Lowe and Eggleston 2009 | To explore “children’s perspectives of an initial assessment at outpatient child and adolescent mental health services (CAMHS).” | New Zealand | 7-12 | Interview | Yes | No | During implementation |
| Morales-Campos et al. 2015 | To describe engagement of “adolescent girls as partners in community-based intervention planning research.” | USA | 11-14 | Participatory retreat, photo mapping | No | Yes | Pre-implementation |
| Muhumuza et al. 2015 | “To understand why the uptake of praziquantel among school children is low and to suggest strategies for improved uptake.” | Uganda | 10-14 | Focus groups | No | Yes | During implementation |
| Nghi et al. 2010 | To present “key findings from formative research conducted in Vietnam to guide human papillomavirus (HPV) vaccine introduction.” | Vietnam | 10-14 | Interviews, focus groups | No | Yes | Pre-implementation |
| Nury et al. 2017 | “To explore children’s perspectives, experiences, and motivations concerning school gardening in order to better understand and increase its potential for health promotion.” | Netherlands | 9-10 | Interviews, participant observation | Yes | Yes | During implementation |
| Okamoto et al. 2014 | To describe “the relevance of a culturally grounded approach toward drug prevention development for indigenous youth populations.” | USA | 11-14 | Focus groups | No | No | Pre-implementation |
| Park et al. 2017 | To test “whether a youth participatory video production program for smoking prevention is feasible and effective.” | USA | mean age 10.95 | Interviews | Yes | No | During implementation |
| Pearson et al. 2012 | To explore “the experiences of children who participated in the Children’s Health and Activity Modification Program.” | Canada | 8-14 | Focus groups | Yes | No | During implementation |
| Pettigrew et al. 2016 | To investigate “consumers’ attitudes to the Tick and its relevance on their purchase decisions.” | Australia | 10-17 | Focus groups | No | Yes | During implementation |
| Pickering et al. 2013 | “To report the views and experiences of children and young people with CP and their families regarding their participation in ADC.” | Wales | 2-18 | Interviews (including with the mosaic method), journaling | No | Yes | During implementation |
| Pittson and Wallace 2011 | “To develop, implement and evaluate a family-based childhood weight management programme.” | England | 11-13 | Focus groups, poster design | No | Yes | Pre-implementation, During implementation |
| Randle et al. 2013 | “To create an educational intervention to induce long-term behavioural change culminating in increased hand hygiene compliance of children, and thus a decrease in the rate of infections.” | England | 5-8 | Focus groups embedded within teaching sessions | Yes | Yes | Both phases |
| Rodriguez et al. 2013 | To assess the “feasibility of a 15-week nutrition education, physical activity, and media literacy program for children living in urban family homeless shelters.” | USA | 9-10 | Participant observation | No | No | During implementation |
| Ross et al. 2016 | “To describe opinions about suicide risk screening in a pediatric medical inpatient sample.” | USA | 10-21 | Interviews | Yes | No | Pre-implementation, During implementation |
| Ruggiero et al. 2015 | “To develop a scalable, sustainable technology-based approach to improve the quality of care in child mental health treatment.” | USA | 8-16 | Interviews, focus groups | Yes | Yes | Both phases |
| Salloum et al. 2015 | “To explore consumers’ and providers’ perceptions of utilizing a CCBT for childhood anxiety in CMHC in an effort to identify factors that may impact implementatoin of CCBT in CMHC.” | USA | 7-13 | Focus groups | No | Yes | During implementation |
| Sanders et al. 2015 | “To examine the efficacy of a multidisciplinary traing-the-trainer model for improving fitness and food label literacy in third grade students.” | USA | 8-9 | Focus groups, drawing | No | Yes | During implementation |
| Sato et al. 2016 | To evaluate “the implementation of the BHCK intervention in recreation centers, and describes lessons learned.” | USA | 10-14 | Open-ended free text boxes on questionnaires/forms | No | No | During implementation |
| Schetzina et al. 2009 | To describe “an initial step in developing a school-based obesity prevention program in rural Appalachia.” | USA | 9-10 | Focus groups | No | Yes | Pre-implementation |
| Sebire et al. 2016 | To report “the main finding from the detailed process evaluation that was conducted (on a RCT of the Bristol Girls Dance Project).” | England | 11-12 | Focus groups | Yes | Yes | During implementation |
| Semple and McCaughan 2013 | To report on “the experience of parental cancer for parents’ and their children and the impact of a psychosocial intervention for young children whose parent has cancer.” | Ireland | 6-11 | Focus groups, drawing | Yes | Yes | During implementation |
| Senior 2014 | “To report on “the planning, implementation and evaluation of an intervention to improve school students’ experience of using the school toilet in a primary school in Melbourne.” | Australia | 6-11 | Focus groups, discussion groups | Yes | No | Both phases |
| Shambley-Ebron 2009 | “To evaluate a culture- and gender-based HIV prevention intervention: My sister, Myself.” | USA | 9-12 | Focus groups, participant observation | No | No | During implementation |
| Sjoberg et al. 2018 | “To examine the feasibility of studying the impact of the Ontario Oncology Nurse School Visitation Program on the well-being and school adjustment of siblings of pediatric cancer patients.” | Canada | 5-13 | Interviews | No | Yes | During implementation |
| Stathi and Sebire 2011 | To evaluate ‘the Y-active, an outreach physical activity and well-being program delived in an inner-city primary school in London, UK by a third-sector partner.” | England | 9-11 | Interviews, focus groups | No | Yes | During implementation |
| Stinson et al. 2012 | “To explore information needs of children with juvenile idopathic arthritis and their parents in order to develop a web-based psychoeducational program aimed at improving their quality of life.” | Canada | 8-11 | Interviews, focus groups | No | Yes | Pre-implementation |
| Subramaniam et al. 2015 | “To highlight the challenges that disadvantaged youth participating in our HackHealth after school program encounter as they identify and articulate their health-related information needs, search for health-related information online, assess the relevance and credibility of this information, and manage and make use of it.” | USA | 10-15 | Interviews, focus groups, participant observation | No | No | During implementation |
| Taylor et al. 2016 | To “report a qualitative evaluation of (“Operation Smoke Storm”) acceptability.” | England | 11-12 | Focus groups | No | Yes | During implementation |
| Temple et al. 2016 | “To describe cycling patterns before and after an innovative learn-to-ride bike camp and factors that facilitate or hinder the generalization of skills developed at camp to home.” | Canada | 7-11 | Interviews | No | Yes | During implementation |
| Tener et al. 2016 | To examine “the subjective experience of children undergoing an invasive examination in the hospital when accompanied by a medical clown.” | Israel | 5-16 | Interviews | No | Yes | During implementation |
| Thomas et al. 2014 | “To explore how parents (a specified target audience) and children (an incidental audience) interpreted and interacted with messages within two major, but very differently framed, anti-obesity social advertising campaigns in Australia.” | Australia | 9-18 | Interviews | Yes | Yes | During implementation |
| Trollvik et al. 2013 | “To explore the participation and responses of children to an asthma education programme that was developed with the aid of children with asthma.” | Norway | 8-10 | Observation | Yes | Yes | During implementation |
| Tunney et al. 2017 | “To explore children’s experience of mindfulness delivered both face-to-face and through a computer game to highlight any differences or similarities.” | Ireland | 10-12 | Focus groups | No | No | During implementation |
| Tutty 2014 | “To present “the results of qualitative research with 116 students (51 boys and 65 girls) ranging from age 6 to 12 who had participated in the Who Do You Tell child sexual abuse education program.” | Canada | 6-12 | Focus groups |  | No | During implementation |
| Tyler and Horner 2008 | “To examine the collaborative negotiation process, an interventional approach being tested at a primary-care school-based clinic to help low-income famiilies improve lifestyle and weight-related health indicators in their overweight children.” | USA | 8-12 | Observation | No | Yes | During implementation |
| Visram et al. 2012 | “To explore the views of key stakeholders in the [’Balance It! Getting the Balance Right’] programme and identify possible reasons for non-completion.” | United Kingdom | 4-16 | Interviews, mosaic methodology- photos, drawings, maps | Yes | Yes | During implementation |
| Waller et al. 2008 | “To pilot an educational programme (KICk-OFF) forchildren and adolescents with type 1 diabetes mellitus.” | United Kingdom | 11-16 | Interviews | No | Yes | During implementation |
| Watson et al. 2012 | To examine “support resources, support-seeking strategies, support and education needs, and intervention preferences of Aboriginal youth with asthma and their caregivers in aneffort to encourage community-wide, health-promoting behaviors.” | Canada | 8-12 | Interviews, drawings, participant observation, sharing circles, focus groups | No | Yes | Pre-implementation |
| Watson-Jones et al. 2015 | To conduct “formative research on facilitators and barriers to HPV vaccination and potential acceptability of a future HPV vaccination programme amongs girls living in hard-to-reach populations in Kenya.” | Kenya | 11-13 | Focus groups | No | Yes | Pre-implementation |
| Westergren et al. 2016 | “To pilot a 6-week exercise intervention designed as active play and examine attendance rate, exercise intensity and children’s perceptions of participation.” | Norway | 10-12 | Focus groups, participant observation | Yes | No | During implementation |
| Willis et al. 2018 | “To describe the association between context, mechanisms and outcome(s) of a participation-focused phyical activity intervention to understand what works, in what conditions, and how.” | Norway | 6-17 | Interviews, focus groups, participant observation | No | Yes | During implementation |
| Woolford et al. 2011 | To explore “participants’ perspectives of... tailored text messages [content] for adolescents enrolled in an existing multidisciplinary weight management program.” | USA | 11-19 | Focus groups | Yes | No | Pre-implementation |
| Wyatt et al. 2013 | “To test OKWAv.2.0’s feasibility and usability (design, interactivity, functionality, and interface) during the development process by using focus groups with 8-year-old to 11-year-old children with asthma.” | USA | 8-11 | Focus groups | Yes | No | Pre-implementation |
